# Evaluation of clinical manifestations, health risks, and quality of life among women with polycystic ovary syndrome

**DOI:** 10.1371/journal.pone.0223329

**Published:** 2019-10-11

**Authors:** Syeda Sidra, Muhammad Haseeb Tariq, Muhammad Junaid Farrukh, Muhammad Mohsin

**Affiliations:** 1 Faculty of Pharmaceutical Sciences, Riphah International University, Islamabad, Pakistan; 2 Department of Clinical Pharmacy, School of Pharmaceutical Sciences, Universiti Sains Malaysia, Penang, Malaysia; 3 Faculty of Pharmaceutical Sciences, UCSI University, Kuala Lumpur, Malaysia; Universita degli Studi dell'Insubria, ITALY

## Abstract

This study aimed to evaluate the clinical manifestations and health risks associated with polycystic ovary syndrome (PCOS) and its impact on quality of life (QOL) in Pakistan. A detailed cross-sectional study was conducted on PCOS among women of reproductive age visiting the gynecology and obstetrics and endocrinology departments at primary and tertiary care hospitals located in Abbottabad, Kohat, and Islamabad. In total, 440 patients meeting the inclusion criteria were included. A checklist was specifically designed to identify symptoms and health risks, including adverse drug reactions, complications, irrational prescription or underprescription, and drug–drug interactions. The Short Form-12 questionnaire was used to evaluate the QOL of patients with PCOS. Data collected were analyzed for descriptive and inferential statistics using chi-square test, analysis of variance, and post hoc analysis. All patients exhibited the cardinal symptoms of PCOS, including obesity (n = 352, 80%), acne (n = 296, 67.3), hirsutism (n = 299, 68%), hyperglycemia (n = 278, 63.2%), and irregular menstruation (n = 316, 71.8%). Ultrasonography confirmed that 268 (61%) patients had multiple cysts of >10 mm in diameter. Patients with untreated PCOS exhibited a high prevalence of health risks including hypertension (n = 87, 19.8%), diabetes (n = 268, 60.9%), sleep apnea (n = 11, 2.5%), infertility (n = 146, 33.2%), increased endometrial thickness (n = 21, 4.8%), miscarriages (n = 68, 15.5%), high cholesterol level (n = 85, 19.3%), and hyperandrogenism (n = 342, 77.7%). Most patients exhibited low QOL scores (n = 374, 85%), with depression being the largest contributor to low QOL. Apart from novel results, this study found an association between depression and low QOL in patients with PCOS, suggesting the need for reviewing the management guidelines and psychological health assessment of women with PCOS.

## Introduction

Polycystic ovary syndrome (PCOS), which affects 6%–20% of women of reproductive age, has been the most debatable female endocrine disorder in the developed world [1], with Pakistan being no exception. It is one of the leading causes of female infertility and is characterized by menstrual irregularities, hirsutism, cystic acne, seborrhea, hair loss, and obesity [2].

The prevalence of PCOS in higher among Pakistani women (52%) than among Western Caucasian women, e.g., 20%–25% in UK [3]. PCOS is associated with significant short- and long-term health problems; these include many metabolic and cardiovascular complications [4,5] as well as psychological disorders [6] such as depression, anxiety, sexual dysfunction, and social problems, which affect a woman’s identity and health-related quality of life (QOL).

The increasingly high incidence of PCOS can be attributed to genetic factors, environmental factors, and intermarriages [7]; however, it is considered an amalgamation of insulin resistance, hyperandrogenemia, and factors causing follicular abnormalities [8]. PCOS has been strongly associated with the future development of type 2 diabetes mellitus, glucose intolerance, hyperinsulinemia, cardiovascular disorders, and hypertension [9,10]. Insulin resistance has been reported in >60% of patients with PCOS [11], including obese and non-obese populations, and 10% of women with PCOS may develop type 2 diabetes by the age of 40 years [8]. Elevated luteinizing hormone levels have been found to be significantly associated with PCOS-associated ovulation disorders and elevated anti-Müllerian hormone (AMH) levels in patients with PCOS [12].

The National Institutes of Health (NIH) diagnostic criteria for PCOS defined in 1990 include hyperandrogenism and oligoovulation but exclude other disorders mimicking PCOS such as adult-onset congenital adrenal hyperplasia, hyperprolactinemia, and androgen-secreting neoplasms [13–16]. However, the Endocrine Society recommends that PCOS should be diagnosed when adult women present with two of the following features: excess androgen production, anovulation, and pearl-sized cysts in the ovaries [17].

PCOS has pronounced effects on the QOL of affected patients. In a multivariate analysis, patients with depression reported low QOL [18]. A randomized controlled trial showed that a stress management program yielded significant reductions in stress and depressive and anxiety symptoms as well as improvements in QOL [19].

Nonetheless, the aspects of PCOS that have a strong influence on affected women remain unclear; thus, it is highly recommended that patient assessment include the evaluation of reproductive and metabolic health and health-related QOL. Various long-term complications and comorbidities have been associated with PCOS, and early diagnosis and therapeutic intervention are warranted in such cases [20]. Moreover, apart from the lack of disease awareness and management as well as medical therapy according to the guidelines, women, mostly in rural areas, are reluctant to visit gynecologists or endocrinologists for treatment despite having symptoms of the disease. This reluctance results in most patients remaining untreated, leading to various future complications. Therefore, the purpose of this study was to investigate the clinical aspects and QOL related to PCOS.

## Materials and methods

### Ethical approval

Ethical approval was obtained from the Ethics Review Board of each participating hospital namely Institutional review board for bioethics, (IRBB), KMU Institute of Medical Sciences Kohat, Dr syed javed hussain Abbottabad diabetes medical and infertility clinic, Dr Farzana Tabbasum Babu shifa doctors clinic, Dr Erum Rubab, PTCL health unit, Islamabad and Professor doctor tanveer Shafqat, Lady Reading Hospital, Peshawar.

Informed consent was obtained from each patient after explaining the research and its objectives. Patients were included only after they signed the informed consent. All researchers ensured patient data confidentiality and compliance with the Declaration of Helsinki.

### Study setting and design

In this prospective, cross-sectional, observational study, patients with PCOS were included who were admitted to any of the following hospitals or who visited their outpatient departments for a routine checkup: Khyber Medical University, Peshawar; KMU Institute of Medical Sciences, Kohat; PTCL Health Center, Islamabad; Abbottabad Diabetes Medical and Infertility Clinic, Abbottabad; Liaqat Memorial Hospital, Kohat; and Bab-ul-Shifa Clinic, Kohat.

### Study sample

The study sample comprised females of reproductive age who met the inclusion criteria based on the NIH 1990 criteria for PCOS and were admitted to or visited the outpatient department of any of the participating hospitals for a routine checkup as well as those who had infertility problems or repeated miscarriages and visited infertility clinics from September 2016 to July 2017. The diagnostic criteria of PCOS on the basis of the NIH 1990 criteria [13–16] include hyperandrogenism and oligoovulation and exclude other disorders mimicking PCOS, such as adult-onset congenital adrenal hyperplasia, hyperprolactinemia, and androgen-secreting neoplasms.

### Inclusion criteria

Females were included in the study based on the following inclusion criteria: (a) reproductive age (15–44 years), (b) history of infertility or repeated miscarriage, and (c) diagnosis of PCOS by the consulting gynecologist/physician based on the NIH 1990 criteria.

### Exclusion criteria

Females were excluded from the study if they met either of the following conditions: (a) age of <15 years or >45 years and (b) no PCOS diagnosis by the consulting gynecologist/physician.

Initially, all patients were diagnosed based on the NIH 1990 criteria as per local practices. The researchers further investigated each patient based on the Rotterdam criteria as well as conducted detailed investigation of menstrual history to identify irregular or absent ovulation and ultrasonography to identify ovarian morphology.

### Sample size

The study sample size was determined using the Epi Info StatCalc software [21,22] and a confidence level of 95%. Accordingly, an approximate sample size of 387 was considered statistically significant. After considering 20% of non-response or missing data values, the final required sample size was determined to be 440.

### Sampling technique

The present study used convenience sampling for patient selection.

### Clinical evaluation

Clinical evaluation of all patients was performed to evaluate improvements in the cardinal symptoms of PCOS, including irregular menstruation, hyperglycemia, obesity, acne, and hirsutism. Furthermore, health risk assessment was performed using data on adverse drug reactions (ADRs), complications, irrational prescribing, underprescribing, and drug–drug interactions. The appropriateness of drug choice and drug–drug interactions were assessed using Physicians’ Desk Reference [23] and Stockley’s Drug Interaction [24].

### Study tool

#### Evidence-based clinical checklist

A thorough and comprehensive review of literature was conducted to develop an evidence-based clinical checklist. Furthermore, variables included in the checklist were compared with those reported in previous studies [25,26] and discussed in a focus group comprising clinical researchers and practitioners. The final developed checklist was also subjected to content validity through a pilot study on 20 patients and to a focus group discussion to identify any overlooked variables or clinical scenarios. After validation, the final version of the checklist was used in the study.

#### QOL assessed using the SF-12 questionnaire

QOL of each patient was determined using the SF-12 questionnaire, which is a 12-point tool comprising mental and physical health-related aspects [27,28]. Responses obtained from each patient were used to calculate the QOL score following the standard method, with low scores indicating poor QOL and vice versa.

### Statistical analysis

All data collected using the clinical checklist and SF-12 questionnaire were recorded in an SPSS spreadsheet after coding and carefully defining all the studied variables. Analysis was then performed in two steps. The first step included descriptive analysis wherein patients’ clinical condition and various variables were analyzed for the respective frequencies, percentages, and measure of central tendency. In the second step, inferential statistics were used to determine the associations among patient- and therapy-related variables, clinical outcomes, and QOL using Pearson’s chi-square test, analysis of variance (ANOVA), and post hoc analysis, with a *p*-value of <0.05 indicating statistically significance.

## Results

In total, 440 patients with PCOS were studied; among these, 274 (62.3%) were aged 15–30 years and 166 (38%) aged 31–44 years. Weight measurements revealed that a large proportion (74.5%) of the patients were normal to nearly obese; approximately, a quarter (24.5%) of the patients were morbidly obese, whereas only a few (0.9%) were underweight. Among the study patients, 364 (82.7%) were married and 76 (17.3%) were unmarried. Table 1 shows that 263 (59.8%) patients had PCOS alone, whereas 177 (40.2%) had PCOS with other gynecological and endocrine disorders.

**Table 1:** Patient demographics (N = 440).

Demographic variables		Frequency, n (%)
**Age (years)**	15–30	274 (62.3)
	31–44	166 (37.7)
**Weight (kg)**	<50	4 (0.9)
	51–100	328 (74.5)
	>101	108 (24.5)
**Marital status**	Unmarried	76 (17.3)
	Married	364 (82.7)
**Major disease**	PCOS	263 (59.8)
	PCOS with other gynecological or endocrine disorders	177 (40.2)

Among the study patients, 278 (63.2%) had hyperglycemia, including 106 (24.1%) with diabetes. Ultrasonography revealed that the majority (n = 268, 61%) of the patients had multiple cysts (diameter > 10 mm) and 34 (8%) had PCOS and endometriosis. Regarding the cardinal symptoms of PCOS, 316 (71.8%) patients had irregular menstruation, 301 (68.4%) had hirsutism, and 296 (67.3%) had acne. Furthermore, depression was reported by 272 (61.8%) patients. Among the comorbidities reported with PCOS, hypertension (7.0%) was the most common, followed by diabetes (22.7%). QOL analysis showed that 374 (85%) patients had poor QOL and 66 (15%) had good QOL (Table 2).

**Table 2:** Clinical characteristics of patients with PCOS.

Clinical characteristics		Frequency, n (%)
**Fasting blood glucose level (mg/dL)**	Normal (<100)	162 (36.8)
	Prediabetes (100–125)	172 (39.1)
	Diabetes (≥126)	106 (24.1)
**Ultrasonography**	Normal	15 (3.4)
	Ovaries with multiple cysts (diameter > 10 mm)	268 (60.9)
	Fibroids	12 (2.7)
	Endometriosis	5 (1.1)
	PCOS and endometriosis	34 (7.7)
	Other pathological conditions	106 (24.1)
**Menstrual flow**	Normal/regular	124 (28.2)
	Amenorrhea	180 (40.9)
	Oligomenorrhea	105 (23.9)
	Menorrhagia	31 (7.0)
**Hirsutism**	Yes	301 (68.4)
	No	139 (31.6)
**Acne**	Yes	296 (67.3)
	No	144 (32.7)
**Depression**	Yes	272 (61.8)
	No	168 (38.2)
**Infertility**	Yes	146 (33.2)
	No	294 (66.8)
**Comorbidity**	Hypertension	31 (7.0)
	Diabetes	100 (22.7)
	Diabetes and hypertension	29 (6.6)
	Cardiac disease	3 (0.7)
	Other endocrine disorder	202 (45.9)
	None	75 (17.0)
**QOL**	Poor	374 (85)
	Good	66 (15)

PCOS, polycystic ovary syndrome; QOL, quality of life

**Fig 1:**
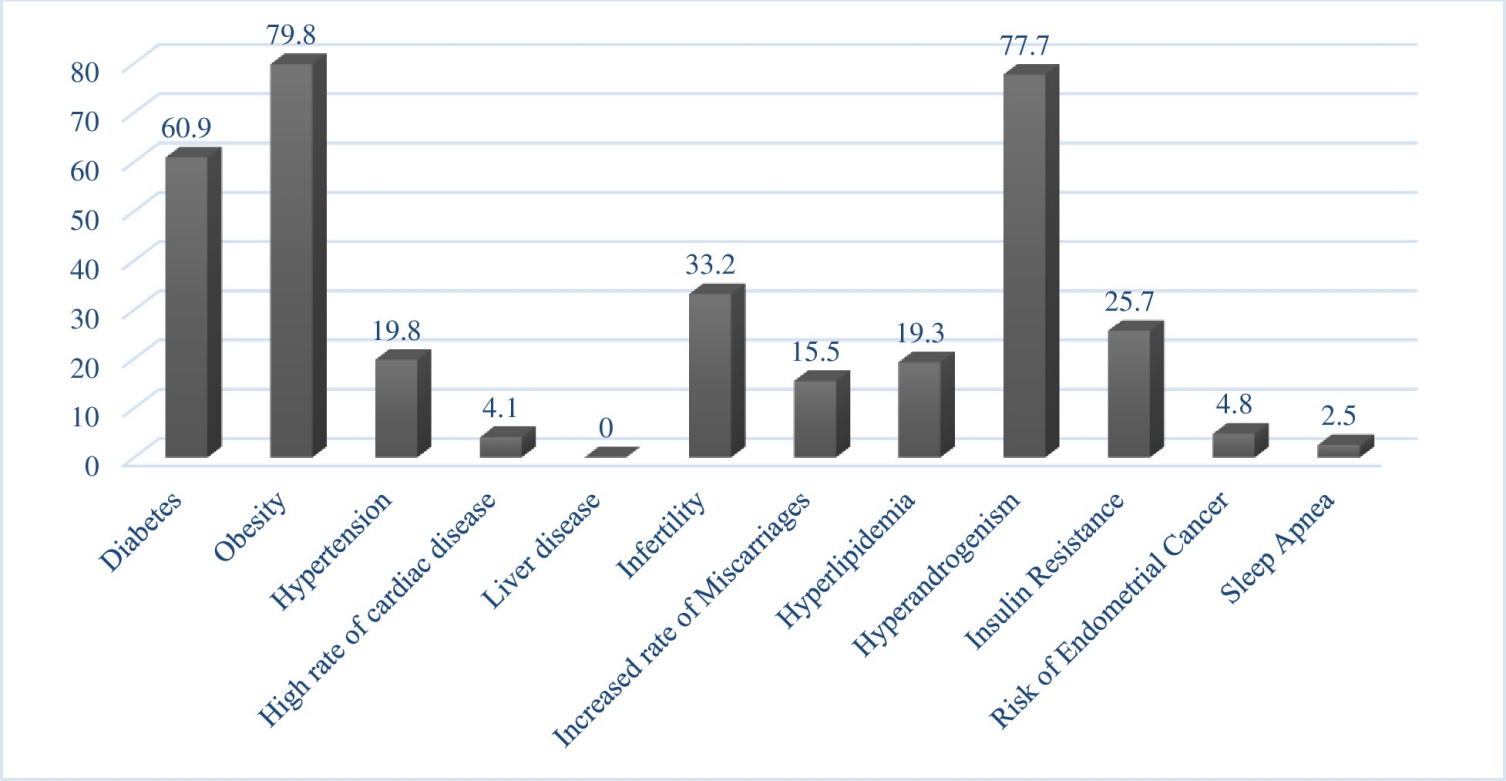
Bar graph showing the percentage of complications among patients with polycystic ovary syndrome.

If not treated properly, PCOS can lead to various serious complications and result in possible aggravation of the syndrome. In the present study, >5 complications were observed in 42 (10%) patients, with the most common complications being obesity (79.8%), hyperandrogenism (77.7%), and diabetes (60.9%). The details of all complications observed in the study population are provided in Fig 1.

Among the 440 patients with PCOS, metformin was prescribed to 358 (81%), contraceptives to 261 (59.4%), infertility drugs to 196 (44.6%), spironolactone to 79 (18%), anti-hirsutism drugs to 21 (4.8%), anti-acne drugs to 34 (7.7%), antihypertensive drugs to 47 (10.7%), anti-obesity drugs to 21 (4.8%), and antidepressant drugs to 35 (8%). Significant drug–drug interactions were observed in 115 (26.1%) patients, which required close monitoring. In addition, the presence of ADRs due the use of these drugs was determined. Accordingly, 433 (98.4%) patients had an ADR rate of >10%, whereas 13 (3%) had an ADR rate of >50%. Detailed description of data regarding prescribed drugs, drug–drug interactions, and ADRs are provided in Table 3.

**Table 3:** Drug-related factors in patients with PCOS.

Drug-related factor		Frequency, n (%)
**Drug–drug interactions**	Significant (closely monitored)	115 (26.1)
	No interaction	325 (73.9)
**Metformin prescribed**	Yes	358 (81.4)
	No	82 (18.6)
**Contraceptives**	Combined oral contraceptives	50 (11.4)
	Progestin only	211 (48.0)
	None	179 (40.7)
**Infertility drugs**	Letrozole	6 (1.4)
	Clomiphene	190 (43.2)
	None	244 (55.5)
**Spironolactone prescribed**	Yes	79 (18)
	No	361 (82)
**Anti-hirsutism drug** **prescribed**	Yes	21 (4.8)
	No	419 (95.2)
**Anti-acne drug prescribed**	Yes	34 (7.7)
	No	406 (92.3)
**Antihypertensive drug** **prescribed**	Yes	47 (10.7)
	No	393 (89.3)
**Anti-obesity drug prescribed**	Yes	21 (4.8)
	No	419 (95.2)
**Antidepressant drug** **prescribed**	Yes	35 (8)
	No	405 (92)
**Percentage of adverse drug reactions in each patient**	<10%	7 (1.6)
	11%–20%	88 (20)
	21%–30%	202 (45.9)
	31%–40%	93 (21.1)
	41%–50%	37 (8.4)
	>50%	13 (3)

PCOS, polycystic ovary syndrome

QOL was determined and analyzed against the cardinal symptoms of PCOS and other variables, with significant differences observed among the disease outcomes; detailed results are presented in Table 4. QOL was found to differ significantly (*p* < 0.05) among patients with menstrual irregularities, hirsutism, acne, obesity, depression, comorbidities, and increased number of complications, including hyperlipidemia, obesity, and hyperandrogenism. Poor QOL scores were observed among patients with menstrual irregularities, hirsutism, acne, hyperglycemia, and obesity. Similarly, poor QOL scores were observed in patients with depression, comorbidities, ovarian cysts, and complications including hyperlipidemia, hyperandrogenism, and insulin resistance.

**Table 4:** Cross-tabulation of various factors affecting QOL.

Variables affecting QOL		QOL		*p*-value
		Poor (n)	Good (n)	
**Menstrual flow**	Normal/regular	107	17	0.044
	Amenorrhea	144	36	
	Oligomenorrhea	93	12	
	Menorrhagia	30	1	
**Hirsutism**	Yes	264	37	0.022
	No	110	29	
**Acne**	Yes	259	37	0.046
	No	115	29	
**Infertility**	Yes	126	20	0.671
	No	248	46	
**Fasting blood glucose level (mg/dL)**	Normal (<100)	133	29	0.409
	Prediabetes (100–125)	150	22	
	Diabetes (>126)	91	15	
**Obesity**	Yes	313	38	0.000
	No	61	28	
**Depression**	Yes	239	33	0.039
	No	135	33	
**Comorbidity**	Hypertension	29	2	0.006
	Diabetes	85	15	
	Diabetes + hypertension	25	4	
	Cardiac disease	3	0	
	Other endocrine disorders	179	23	
	None	53	22	
**Ultrasonography**	Normal	14	1	0.230
	Ovaries with multiple cysts (diameter > 10 mm)	220	48	
	Fibroids	12	0	
	Endometriosis	5	0	
	PCOS and endometriosis	31	3	
	Other pathological condition	92	14	
**Percentage of adverse drug reactions in each patient**	<10%	7	0	0.285
	11%–20%	70	18	
	21%–30%	169	33	
	31%–40%	82	11	
	41%–50%	34	3	
	>50%	12	1	
**Number of complications**	<5	290	59	0.082
	5	46	3	
	>5	38	4	
**Hyperlipidemia**	Yes	66	19	0.042
	No	308	47	
**Hyperandrogenism**	Yes	305	37	0.000
	No	69	29	
**Insulin resistance**	Yes	100	13	0.285
	No	274	53	
**Risk of endometrial cancer**	Yes	16	7	0.063
	No	358	59	

QOL, quality of life

**Fig 2:**
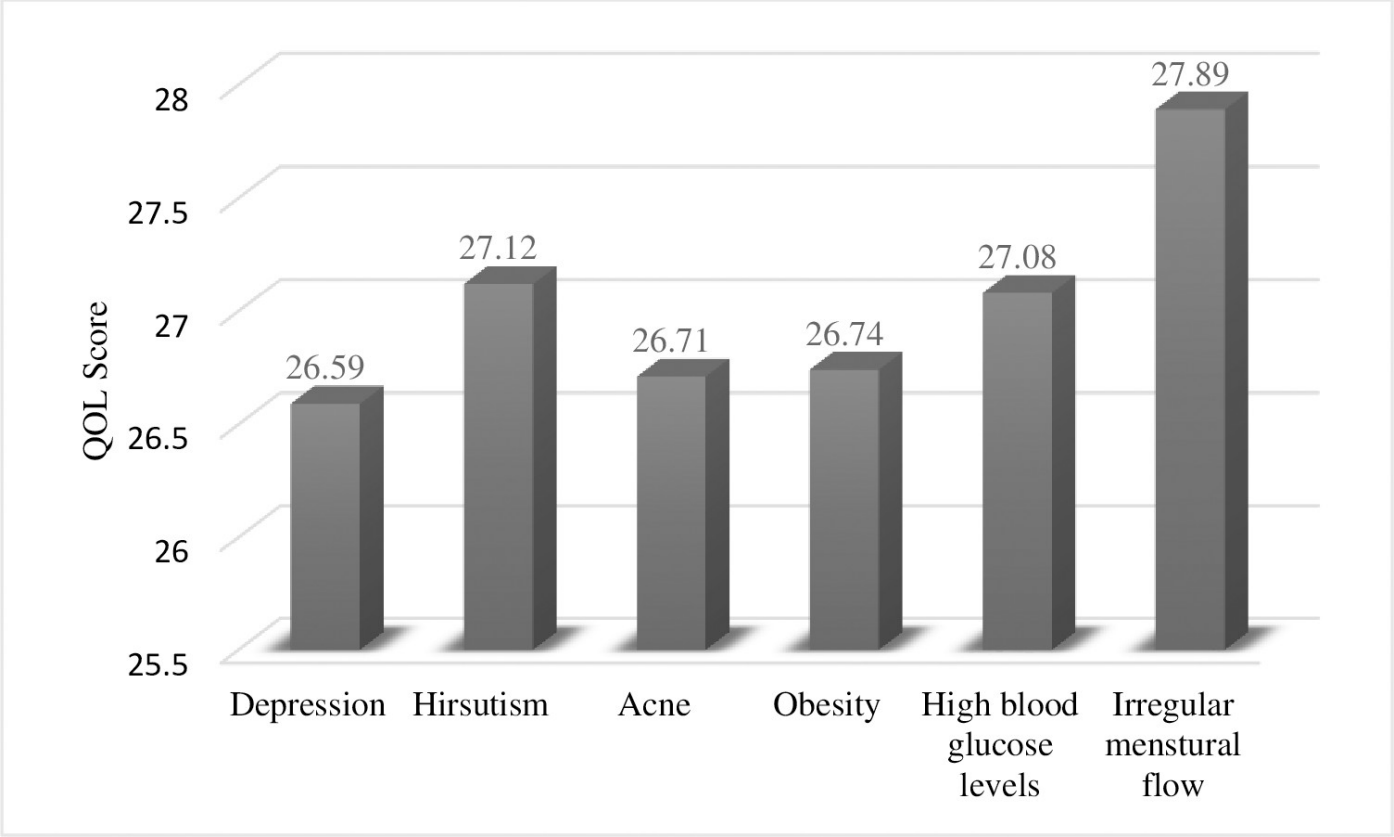
Bar graph showing the mean QOL scores according to clinical conditions in patients with PCOS. QOL, quality of life; PCOS, polycystic ovary syndrome.

After analyzing the mean QOL scores according to the clinical conditions associated with PCOS, it was observed that depression was the largest contributor to low QOL among patients with PCOS, followed by acne and obesity. The mean QOL scores are presented in Fig 2.

The association between ADRs and prescribed drug use is shown in Table 5. The table presents the major ADRs associated with prescribed drug use. The most common ADR was diarrhea, which was observed in 81.4% of the patients, followed by nausea in 65.9%, mood changes in 64.1%, and abdominal pain and breast tenderness in 59.3%. A significant difference (*p* < 0.05) was observed in terms of the occurrence of diarrhea with metformin and clomiphene use; abdominal pain with metformin, combined oral contraceptive (COC), clomiphene, and letrozole use; and breast tenderness with clomiphene, letrozole, and spironolactone use. Metformin was found to significantly affect the occurrence of diarrhea, vomiting, abdominal pain, dysuria, hot flashes, and reduced libido. Progestin was found to be associated with vaginal/uterine bleeding, dysuria, reduced libido, weight changes, headaches, and confusion. COCs were most commonly associated with abdominal pain, nausea, mastalgia, hot flashes, reduced libido, and mood changes. Clomiphene significantly affected the occurrence of diarrhea, vomiting, abdominal pain, vaginal/uterine bleeding, breast tenderness, mastalgia, hot flashes, headaches, and confusion. Letrozole significantly affected the incidence of abdominal pain, breast tenderness, dysuria, weight changes, mood changes, and headache. Further, spironolactone significantly affected the occurrence of vaginal/uterine bleeding, breast tenderness, dysuria, reduced libido, mood changes, and headaches.

**Table 5:** Association between adverse drug reactions and prescribed drug use.

ADR	Yes, n (%)	No, n (%)	Metformin *P*-value	Progestin *P*-value	COC *P*-value	Clomiphene *P*-value	Letrozole *P*-value	Spironolactone *P*-value
**Diarrhea**	358 (81.4)	82 (18.6)	0.000	0.218	0.112	0.000	0.689	0.281
**Vomiting**	89 (20.2)	351 (79.8)	0.020	0.517	0.569	0.008	0.255	0.449
**Abdominal pain**	261 (59.3)	179 (41)	0.001	0.325	0.000	0.002	0.043	0.462
**Nausea**	290 (65.9)	150 (34)	0.441	0.312	0.000	0.224	0.081	0.264
**Vaginal/uterine bleeding**	82 (18.6)	358 (81)	0.293	0.000	0.847	0.001	0.599	0.036
**Breast tenderness**	261 (59.3)	179 (40.7)	0.100	0.200	0.522	0.006	0.043	0.007
**Mastalgia**	142 (32.3)	298 (67.7)	0.295	0.065	0.000	0.021	0.095	0.144
**Dysuria**	179 (40.7)	261 (59.3)	0.000	0.046	0.193	0.292	0.043	0.000
**Hot flashes**	126 (28.6)	314 (71.4)	0.000	0.204	0.023	0.000	0.130	0.508
**Reduced libido**	137 (31.1)	303 (69.9)	0.000	0.022	0.047	0.224	0.105	0.018
**Weight changes**	197 (44.8)	243 (55.2)	0.176	0.000	0.117	0.132	0.027	0.100
**Mood changes**	282 (64.1)	158 (35.9)	0.312	0.090	0.008	0.227	0.002	0.000
**Headaches**	215 (48.9)	225 (51)	0.265	0.036	0.374	0.000	0.013	0.001
**Confusion**	143 (33)	297 (68)	0.289	0.013	0.538	0.004	0.093	0.226

ADR, adverse drug reaction; COC, combined oral contraceptive

Table 6 describes the appropriateness of administered drug therapy for the management of PCOS symptoms and complications and its association with QOL. It was observed that various symptoms and complications were left untreated, which led to reduced QOL scores. Accordingly, 32.9% of the patients with infertility remained untreated; thus, all these patients had poor QOL. Menstrual irregularities remained untreated in 109 (34.5%) patients, among whom 103 had poor QOL. Hirsutism and acne remained untreated in 286 (95%) and 262 (88.5%) patients, among whom 249 and 225 patients had poor QOL, respectively. Hyperandrogenism remained untreated in 273 (79.8%) patients, among whom 248 had poor QOL. Depression, obesity, and hypertension were not appropriately managed in 247 (90.8%), 330 (94%), and 43 (49.4%) patients, among whom 215, 293, and 35 patients had poor QOL, respectively. Significant associations were determined using chi-square test (using nominal scale QOL scores such as poor and good) and one-way ANOVA (using QOL score of each patient). Chi-square test showed that QOL scores significantly differed (*p* < 0.05) among patients taking infertility drugs, contraceptives, anti-acne drugs, and metformin. Meanwhile, one-way ANOVA revealed that QOL scores significantly differed (*p* < 0.05) among patients taking infertility drugs, anti-hirsutism drugs, anti-acne drugs, metformin, and antidepressants.

**Table 6:** Appropriateness of administered drug therapies for clinically significant conditions in patients with PCOS.

Clinical condition	Appropriate therapy provided		Quality of life		*p*-value
	Response	n (%)	Poor	Good	
**Infertility**	Yes	98 (67.1)	78	20	0.000^a^
	No	48 (32.9)	48	0	0.020^b^
**Abnormal menstrual flow**	Yes	207 (65.5)	164	43	0.000^a^
	No	109 (34.5)	103	6	0.818^b^
**Hirsutism**	Yes	15 (5)	15	0	0.230^a^
	No	286 (95)	249	37	0.048^b^
**Acne**	Yes	34 (11.5)	34	0	0.000^a^
	No	262 (88.5)	225	37	0.026^b^
**Hyperglycemia**	Yes	250 (89.9)	215	35	0.009^a^
	No	28 (10.1)	26	2	0.001^b^
**Hyperandrogenism**	Yes	69 (20.2)	57	12	0.080^a^
	No	273 (79.8)	248	25	0.752^b^
**Depression**	Yes	25 (9.2)	24	1	0.332^a^
	No	247 (90.8)	215	32	0.000^b^
**Obesity**	Yes	21 (6)	20	1	0.714^a^
	No	330 (94)	293	37	0.131^b^
**Hypertension**	Yes	44 (50.6)	38	6	0.572^a^
	No	43 (49.4)	35	8	0.811^b^

^a^Chi-square test

^b^One-way analysis of variance

**Fig 3:**
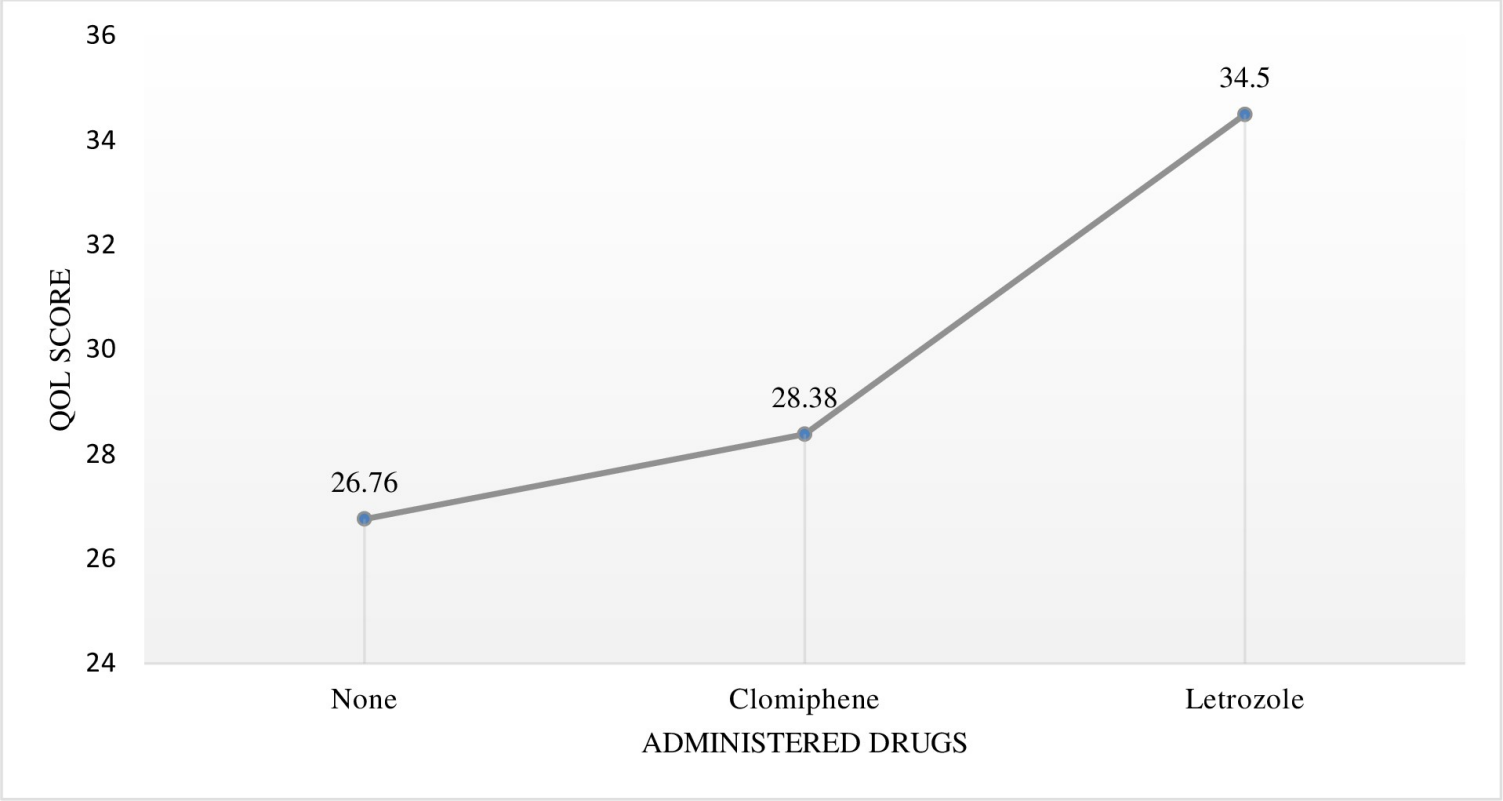
Line graph showing the comparison of the mean quality of life (QOL) scores among patients receiving infertility drugs.

In the present study, 33.2% of the patients had infertility; among these, 67.1% were administered clomiphene and letrozole. To determine differences in terms of mean QOL scores between patients receiving clomiphene and those receiving letrozole, one-way ANOVA and Tukey’s post hoc analysis were performed. The results of the post hoc analysis are summarized in Table 7, and the comparison between mean scores is presented in Fig 3.

**Table 7:** One-way ANOVA and post hoc analysis of differences in terms of QOL scores between patients receiving clomiphene and those receiving letrozole.

One-way ANOVA						Tukey’s post hoc analysis		
	Sum of squares	df	Mean square	F	Sig.	Infertility drugs		Subset for alpha 0.05
**Between groups**	574.902	2	287.451	4.423	0.013	None	26.76	
**Within group**	28403.186	437	64.996			Clomiphene	28.38	
**Total**	28978.089	439				Letrozole		34.50

ANOVA, analysis of variance; QOL, quality of life; df, degrees of freedom; F; Sig.

One-way ANOVA confirmed a significant difference (*p* < 0.05) in terms of QOL scores between patients receiving clomiphene and those receiving letrozole. Post hoc analysis revealed differences in terms of mean scores and showed that patients receiving letrozole had higher QOL scores than those receiving clomiphene.

## Discussion

### Study overview

The present study documents the impact of PCOS and its complications on QOL and comprehensively analyzed prescribing practices followed at primary and tertiary healthcare centers in Pakistan.

A total of 440 patients of reproductive age visiting primary and tertiary healthcare centers across Pakistan participated in this study. Patients were categorized according to their age, weight, marital status, and major presenting disease; subsequently, their association with different variables was studied. Accordingly, 62.3% of the patients were aged 15–30 years, indicating a higher prevalence of PCOS among younger patients. The differences in clinical outcomes and quality of life among patients of various age groups were found to be statistically insignificant.

All participants had ≥1 complication of PCOS, with the most common complication being obesity (80%), followed by hyperandrogenism (77.7%), diabetes (60.9%), and infertility (33.2%). The diverse features of PCOS make it difficult to analyze affected patients and to compare data with those reported in other national or international studies. The management of PCOS in obese patients is difficult, and if left untreated, it may give rise to future risk factors described earlier in the present study.

### Treatment of PCOS and its complications and their impact on QOL

The first-line treatment for PCOS has been lifestyle interventions; however, major clinical features such as acne, hirsutism, menstrual irregularities, and anovulation require pharmacological treatment that includes contraceptives, such as COC and progestin-only contraceptives, and infertility drugs, such as letrozole or clomiphene, metformin, and spironolactone. More drugs need to be added to control these complications and various signs and symptoms, such as acne, hirsutism, obesity, hypertension, hyperlipidemia, depression, and anxiety. The use of metformin is important for enhancing menstrual cycle regulation, ovulation rate, and chances of pregnancy in patients facing difficulties in weight loss. In the present study, it was found that metformin was used alone or in combination with clomiphene citrate (an anovulatory drug). According to a randomized, controlled trial conducted in Pakistan, a combination of metformin and simvastatin more effectively managed PCOS than metformin alone [29].

Accumulating evidence from several studies suggests that insulin resistance is one of the most important mechanisms underlying the pathology of PCOS. One study conducted in Pakistan revealed a higher frequency of hyperinsulinemia (fasting insulin level > 10) and insulin resistance relative to the rates reported in studies on white Caucasian women with PCOS [30]. Accordingly, the use of insulin sensitizers such as inositol isoforms, which have a good safety profile and effectiveness, has gained increasing attention. Inositol has been proven to be effective in patients with PCOS because they improve metabolic and hormonal states and restore spontaneous ovulation. In assisted reproductive technology, inositol has been shown to improve the parameters of ovarian stimulation [31]. Another study evaluated the metabolic and hormonal effects of combined myo-inositol and d-chiro-inositol therapy on patients with PCOS. Notably, the authors observed a significant decrease in body weight and fT, follicle-stimulating hormone (FSH), LH, and insulin levels as well as a significant increase in the serum SHBG level. Moreover, the serum glucose level in OGTT decreased after 6 months of treatment and skin conditions improved after only 3 months of treatment [32].

PCOS can be considered a consequence of concurrent and inter-related endocrine alterations. Recent data suggest that hyperinsulinemia and insulin resistance are of paramount importance in the development of hyperandrogenism, which leads to anovulation. Accordingly, it is not surprising that MI and DCI yielded excellent effects on metabolic and hormonal parameters in patients with PCOS [33]. The existing data regarding drug metabolism in patients with PCOS appear to be extremely limited. This important knowledge gap could have significant implications for therapeutic approaches and future perspectives because the dosages of drugs commonly used to treat PCOS should be tailored according to each patient’s characteristics [34]. A study evaluated the potential application of a nomogram based on a woman’s age and ovarian reserve markers for optimizing the initial FSH dose during intrauterine insemination (IUI) cycles. Notably, the nomogram-calculated FSH starting dose was significantly lower than the actual prescribed dose (*p* < 0.001) in most cases, whereas the nomogram-calculated FSH starting dose was significantly higher than the actual prescribed dose in only 14.8% of IUI cycles. The application of nomogram during IUI cycles would enable more tailored FSH starting doses and improved cost-effectiveness [35].

The aforementioned drugs have been proven efficacious for treating symptoms and improving QOL albeit with many adverse effects, affecting patients’ physical health. The most profound adverse effect was diarrhea, which was found in almost 80% of the patients, followed by nausea (66%), mood changes (64%), breast tenderness or pain (60%), abdominal pain (59%), and back pain (43.6%). This suggests that the majority of drugs exert gastrointestinal adverse effects. Although metformin, Clomid, and Provera were associated with diarrhea, worse symptoms were observed with metformin in some patients. Nonetheless, the adverse effects of metformin can be minimized by lowering drug doses and taking it with meals.

In general, all available treatments were associated with a low incidence of adverse effects and appeared to be well tolerated. The results presented herein are well supported by a similar meta-analysis on the adverse effects of common PCOS treatments [36]. PCOS has been associated with long-term risk factors such as cardiac diseases, type 2 diabetes mellitus, infertility, sleep apnea, high cholesterol levels, endometrial carcinoma, and insulin resistance, leading to serious complications if left untreated; these risk factors have been identified herein. The occurrence of some major risk factors reported in the present study is consistent with previously reported findings [37].

Dietary restriction and fasting have become increasingly popular considering their potential modifying effects on metabolism and possibly the more durable outcomes of epigenetic modulation. Under fasting conditions, the metabolic imbalance characteristic of PCOS may show significant improvement. Different fasting regimens can reduce IGF-1, IGFBP1, glucose, and insulin levels and consequently have beneficial effects on ovarian function, androgen excess, and infertility in women with PCOS [38]. One study conducted in Iran evaluated the effects of Ramadan fasting on the metabolic statuses of women with PCOS and found that this 4-week fasting had beneficial effects on nitrous oxide and plasma glutathione levels but did not affect glucose hemostasis parameters, lipid profiles, or total antioxidant capacity [39]. This result is attributed to the fact that not all women with PCOS exhibit glucose metabolism abnormalities. This might also be explained by the complex relationship among insulin resistance, glucose homeostasis, hormonal balances, and lipid concentrations [40].

Our results show that various symptoms and complications were left untreated, which led to reduced QOL scores.

#### Depression and anxiety

Among the study patients, 61.8% had depression, the prevalence of which was high among young married patients. Depression was found to affect not only psychological wellness but also physical health. PCOS was managed in only 74.6% of the patients with depression. Similarly, a study conducted in Pakistan reported higher depression and anxiety scores in patients with PCOS who exhibited increases in insulin resistance and lipid profiles [41]. Moreover, poor QOL was observed in 87.8% of these patients and only 9.2% of them received treatment for PCOS. No significant difference was found in QOL even with antidepressant treatment. The lack of improvement in QOL can be linked to the coexistence of acne (86%; *p* < 0.05), hirsutism (83%, *p* < 0.05), and infertility (30%, *p* < 0.05), which can lead to severe depression [42]. The prevalence of depression among the present study patients is relatively similar to that reported by Barnard et al. (67%) [43]. The same study showed that patients with PCOS and depression had lower QOL scores than those without depression [43]. However, unlike our study, they did not determine the impact of depression therapy on QOL.

#### Hirsutism

Our results showed that 68.4% of the patients had hirsutism; among these, 87% had poor QOL (*p* < 0.05). The majority of the patients had not been administered any treatment for hirsutism despite its significant effect on QOL. Accordingly, only 4.8% of the patients received the recommended therapy for hirsutism, which did not improve QOL. These results are similar to those reported in a study conducted in Iran wherein multivariate analysis revealed that hirsutism had the strongest impact on QOL (*p* < 0.001) [44].

#### Infertility

In addition to being the most common issue among patients with PCOS, infertility significantly affects QOL scores. In the present study, 33.2% of the patients had infertility; among these, 67.1% received treatment comprising clomiphene and letrozole. One-way ANOVA confirmed a significant difference (*p* < 0.05) in terms of QOL scores between patients receiving clomiphene and those receiving letrozole. Post hoc analysis, which was used to determine the differences in mean scores, showed that patients receiving letrozole had higher mean QOL scores than those receiving clomiphene. Although previous guidelines have recommended the use of clomiphene for the management of infertility, recent guidelines have suggested the use of aromatase inhibitors such as letrozole because it has been shown to increase pregnancy rates by 40% with a shorter time to pregnancy [45]. Similarly, a study involving patients with PCOS found that the use of letrozole for ovulation induction had a better effect on the levels of endometrial receptivity markers than the use of clomiphene citrate [46].

#### Obesity

The overall prevalence of obesity was found to be 80%. Among the patients with obesity, 62% were from the younger age group and 38% from the older age group, although no significant difference was observed between the two groups (*p* > 0.05). Our study found that younger patients had higher rates of obesity as a complication of PCOS, which has never been reported previously. Studies have reported increased incidence of obesity among patients with PCOS with a significant increasing trend with age (*p* < 0.001) [47]. In the older age group, obesity has also been associated with age-related metabolic disorders. Given that the prevalence of PCOS was higher among younger patients, the complications of obesity were also higher in them than in older patients.

Lower SF-12 scores, which indicate a low QOL, were mostly found among patients with obesity, hirsutism, and acne. The finding that weight issues had the most negative impact on health-related QOL is not surprising considering the overwhelmingly difficulty in losing weight without surgery faced by morbidly obese patients. Accordingly, high rates of weight regain have been observed and weight loss interventions have mostly been unsuccessful. Due to the apparent inability of patients with PCOS to lose weight, some reported frustration. This has also been discussed in a similar study investigating the effect of obesity on QOL [37]. In the present study, depression was found to be the largest contributor to low QOL, followed by acne and obesity (*p* < 0.05). This finding can be considered unique given that depression has never been reported to be associated with the lowest QOL score and previous studies have found obesity to be the largest contributor to low QOL scores [43]. Considering the present findings regarding obesity in younger age groups and its possible association with acne and hirsutism, future studies could seek to determine the impact of anti-acne, anti-hirsutism, and antidepressant therapies on QOL.

## Strengths and limitations of the study

The strengths of the current study include a large, multi-centric patient population, detailed study of all relevant clinical aspects and conditions, and research focus on the clinical and statistical significance of each variable. Moreover, this study is unique because it developed an association of psychometric assessment including QOL with clinically significant conditions. The information collected during a literature review revealed that most currently available studies lack these strengths.

The major limitation of the present study is its cross-sectional design, which did not enable a longitudinal study of the long-term effects of drugs and various complications. Another limitation of this study is the lack of adequate financial resources to determine the levels of various significant biochemical parameters, including those of AMH, androgens, triglycerides, high-density lipoproteins, and glucose tolerance. Furthermore, the body mass index could not be determined due to a lack of patient height data. All patients were declared obese/overweight according to their body weight as per the local practices of the participating hospitals.

## Conclusions

Patients with PCOS exhibit poor QOL, which is associated with depression, acne, and hirsutism. Therefore, PCOS management guidelines should review the recommendations regarding the use of pharmacological agents for these conditions. The clinical conditions and complications associated with PCOS should be given due importance while selecting appropriate management plans for each patient considering the effects of these conditions on overall morbidity and QOL.

## Supporting information

S1 File Data sets of the study. (XLSX)
